# Analysis of IL-6, STAT3 and HSPA1L Gene Polymorphisms in Anti-Tuberculosis Drug-Induced Hepatitis in a Nested Case-Control Study

**DOI:** 10.1371/journal.pone.0118862

**Published:** 2015-03-19

**Authors:** Jing Wang, Ru Chen, Shaowen Tang, Xiaozhen Lv, Shanshan Wu, Yuan Zhang, Yinyin Xia, Pei Gao, Dehua Tu, Dafang Chen, Siyan Zhan

**Affiliations:** 1 Department of Epidemiology and Biostatistics, School of Public Health, Peking University Health Science Centre, Beijing, China; 2 Department of Epidemiology and Biostatistics, School of Public Health, Nanjing Medical University, Nanjing, China; 3 Clinical Research Division, Peking University Institute of Mental Health, and Key Laboratory for Mental Health, Ministry of Health, Beijing, China; 4 Department of Clinical Epidemiology and Biostatistics, McMaster University, Hamilton, Canada; 5 Center for Tuberculosis Control and Prevention, Chinese Center for Disease Control and Prevention, Beijing, China; 6 Department of Tuberculosis Treatment, Beijing Institute for Tuberculosis Control, Beijing, China; Nanjing Medical University, CHINA

## Abstract

**Objectives:**

To investigate the association of IL-6, STAT3 and HSPA1L polymorphisms with the risk of anti-tuberculosis drug-induced hepatitis (ATDH) in Chinese Han population.

**Methods:**

The study was designed as a nested case-control study within a prospective cohort. Each case was matched with four controls by sex, age at baseline (±5 years), treatment history, disease severity, drug dosage and place of sample collection. Genetic polymorphisms of IL-6, STAT3 and HSPA1L were determined blindly by TaqMan single-nucleotide polymorphism (SNP) genotyping assay. Odds ratio (OR) with 95% confidence intervals (CIs) was estimated by conditional logistic regression model to measure the association between selected SNPs and the risk of ATDH.

**Results:**

A total of 89 incident ATDH cases and 356 ATDH-free controls were genotyped for IL-6 (rs2066992, rs2069837, rs1524107), STAT3 (rs1053004, rs1053023, rs1053005) and HSPA1L (rs2227956). In genotype analysis, no significant difference was observed in genotypes frequencies of the seven selected SNPs between case and control group after Bonferroni correction. In haplotype analysis, carriers with STAT3 GAT and AGC (rs1053023-rs1053005-rs1053004) haplotypes had a significantly higher risk of ATDH compared with wild-type haplotype (P<0.0001).

**Conclusion:**

This study suggested that genetic variants of STAT3 might contribute to ATDH susceptibility in Chinese Han population. Studies in larger, varied populations are required to confirm these findings.

## Introduction

Tuberculosis (TB) remains a major global health problem, causing ill-health among millions of people each year and ranking as the second leading cause of death from infectious diseases worldwide. According to World Health Organization (WHO) report, there were 8.6 million new cases in 2012 and China alone accounted for 12% of total cases behind India [1].

In order to control TB epidemic, directly observed treatment strategy (DOTS) therapy has been implemented in China, which is a standard chemotherapy regimen requiring continually taking drug combinations of isoniazid (INH), rifampicin (RIF), pyrazinamide (PZA), ethambutol (EMB) and/or streptomycin (SM) for six to nine months [2]. Although a vast majority of patients tolerate the drugs, the outcome may be less favorable in those with undesirable adverse drug reactions (ADRs). Anti-tuberculosis drug-induced hepatitis (ATDH) as one of the most serious ADRs would not only increase the suffering of the patients, but also cause incompliance, drug resistance and even treatment failure [3,4]. Therefore, efforts to enhance the understanding of the risk factors and pathogenesis of ATDH are urgently needed.

Risk factors associated with ATDH include female sex, older age, pre-existing liver disease, heavy alcohol consumption, and HIV or hepatitis B or C virus co-infection [5]. In addition, genetic factors contributing to the susceptibility of ATDH involving genetic variations in drug metabolism, drug transport, acquired immune responses have been studied extensively [6,7,8,9]. However, the the roles of cytokines in ATDH still remain largely uncharacterized.

Limited studies have investigated the association between cytokine genetic polymorphisms and the risk of drug induced liver injury (DILI). Aithal [10] reported that the frequencies of the variant alleles for IL-10 and IL-4 were significant higher in patients with diclofenac-induced hepatitis compared with both healthy controls and subjects on diclofenac without hepatitis. Kim [11] revealed that TNF-∝ polymorphism-308G/A was significantly associated with ATDH (OR = 1.95 95% CI 1.11–3.14). Liang [12] found that IL-10–592 AA and IL-10–819 TT genotypes significantly increased the incidence of DILI in breast cancer patients. However, controversial results were reported in another study [13]. Taken together, all these studies suggested that cytokine genetic polymorphisms may contribute to the risk of DILI.

Among the numerous cytokines relevant to DILI susceptibility and progression, there is compelling evidence that Interleukin-6 (IL-6) plays an important role in acute phase response, liver regeneration and protection against liver injury by a variety of drugs [14,15,16]. It is also well known that the protective effect of IL-6 is mediated through IL-6/STAT3 signaling pathway which provides hepatoprotection against Fas and toxic damage by direct inactivation of caspases and reduction of reactive oxygen species [17,18]. Previous study also found that IL-6 may protect against DILI by up-regulating the heat shock protein (HSP) expression [19], and HSPs protect against apoptosis, necrosis [20] and toxin insult [21]. HSPs are a group of conserved proteins expressed ubiquitously in cells and the HSP70 family (70kD HSP) is probably the most predominant among all HSP proteins [22]. Some experimental studies have proved the liver protection role of HSP70 genes against hepatotoxic agents by protecting mitochondria and interfering with the stress-induced apoptotic program [23,24]. Genetic association studies also found that HSP70 polymorphisms were associated with many diseases such as hepatocellular cancer, breast cancer, gastric cancer and Crohn’s disease in Chinese population [25,26]. Therefore, we hypothesized that IL-6/STAT3 pathway and the downstream HSP70 responsiveness may contribute to the IL-6-mediated protective effect against DILI.

To our knowledge, there was only one case-control study with 37 patients and 32 controls found that IL-6 polymorphisms/haplotypes were associated with the risk of tacrine-induced liver injury [27]. And one GWAS study [28] in patients taking flucloxacillin observed a significant difference between cases and controls in HSPA1L rs2227956, however these associations have not been investigated in ATDH patients. And there is no published study investigating the association of genetic variants of STAT3 gene with susceptibility of ATDH.

This study is the first attempt aiming to investigate the association of IL-6, STAT3 and HSPA1L polymorphisms with the risk of ATDH in Chinese Han population.

## Materials and Methods

### Study population

This case-control study was nested in a cohort study entitled *Anti-tuberculosis Drugs Induced Adverse Reactions in China National Tuberculosis Prevention and Control Scheme Study* (ADACS) [29]. This study was approved by the Ethics Committee of Center for Tuberculosis Control and Prevention of China and Health Science Center of Peking University. Written informed consent was obtained from every participant or surrogate before enrolment. From October 2007 to June 2008, a total of 4488 newly diagnosed patients with sputum smear positive pulmonary TB were recruited from four provinces (Zhejiang, Guangxi, Chongqing, Jilin) in China. Some characteristics of this cohort have been reported previously [30,31]. Patients were excluded for the following reasons: 1) abnormal serum ALT, AST or total bilirubin levels before anti-TB treatment; 2) positive serological testing for hepatitis B virus; 3) concomitant use of hepatotoxic drugs or regular alcohol intake; 4) history of liver disease or systemic diseases that may cause liver dysfunction.

All primary/retreatment patients with pulmonary TB were treated with a combination regimen including INH (600 mg), RIF (600 mg, or 450 mg if body weight was < 50 kg), PZA (2000 mg) and EMB (1250 mg) for the first two months (retreatment patients were injected with SM 750 mg each time simultaneously) and then with the same regimen, without PZA and EMB, for another four months for primary patients and with the same regimen, without PZA and SM, for another six months for retreatment patients. Doses did not vary with ages and sex. Before anti-tuberculosis treatment, serum hepatitis B virus surface antigen (HBsAg), serum alanine transaminase (ALT), aspartate amino transaminase (AST), direct and total bilirubin levels, renal function, blood and urine routine test were measured and these tests were conducted again within two months after the onset of treatment or whenever patients had symptoms of suspected hepatitis (such as anorexia, nausea, vomiting, malaise, tea-colored urine) [32].

ATDH was defined as: 1) an increase in ALT level greater than two-times of the upper limit of normal (ULN) with/without a combined increase in AST and total bilirubin levels provided one of them was greater than two-times of ULN during the treatment [33]; 2) causality assessment result was certain, probable or possible based on the WHO Uppsala Monitoring Center system [34]. All suspected hepatitis patients were reviewed by experts from Chinese State Food and Drug Administration. Patients who fulfilled the criteria of ATDH were assigned into the case group, and each case was matched with four ATDH-free controls by sex, age at baseline (±5 years), treatment history (categorized as primary treatment patients and retreatment patients), disease severity (based on the range of lesions on X-ray films and categorized as minimal, moderately and far advanced on the basis of the National Tuberculosis Association of USA [35]), drug dosage and place of sample collection.

### SNPs selection

For the replication purpose, the public SNP database (NCBI dbSNP) and Pubmed were searched to identify previously reported genetic polymorphisms of candidate gene IL-6, STAT3 and HSP70 in association with DILI. Then, SNPs were selected if the minor allele frequencies (MAF) in Chinese Han population was > = 0.10. As a result, three SNPs were selected which were rs2066992 and rs2069837 for IL-6 and rs2227956 for HSPA1L (Table 1).

**Table 1:** Information of seven selected SNPs.

Gene	SNP No.	Chromosome Position^a^	Location	Base Change	MAF ^b^ (%)	HWE P-value ^c^
IL-6	rs2066992	22768249	Intron 7	G>T	24.4%	0.322
	rs2069837	22768027	Intron 7	A>G	19.8%	0.089
	rs1524107	22768219	Intron 7	C>T	24.4%	0.252
STAT3	rs1053004	40466092	3’UTR	T>C	32.6%	0.630
	rs1053023	40465616	3’UTR	A>G	27.9%	0.657
	rs1053005	40465910	3’UTR	A>G	26.7%	0.588
HSPA1L	rs2227956	31778272	Missense	T>C	18.6%	0.235

^a^ SNP position in NCBI dbSNP (http://www.ncbi.nlm.nih.gov/projects/SNP).

^b^ Minor allele frequency (MAF) for Han Chinese in Beijing in the HapMap database (http://www.hapmap.org).

^c^ Hardy—Weinberg equilibrium (HWE) P-value in the control group.

For gene STAT3, as there is no report previously, SNPs were selected based on tag SNP (tSNP). For IL-6 gene, as the selected two SNPs were not functional SNPs, we also select SNPs based on tSNP approach as a supplement. All eligible SNPs including 2kb upstream and downstream of STAT3 were downloaded from the Chinese Han population (CHB) database of HapMap (http://www.hapmap.org/). Then, tSNPs were selected using Haploview 4.2 software according to the following criteria: 1) MAF > = 0.10; 2) r^2^ of pairwise linkage disequilibrium > = 0.8. As a result, three SNPs (rs1053004, rs1053023, rs1053005) of STAT3 and three SNPs (rs2066992, rs2069837, rs1524107) of IL-6 were selected for genotyping (Table 1).

### Genotyping

Genomic DNA was extracted from blood. Genotyping was performed by TaqMan allelic discrimination technology on the ABI 7900 Real-Time PCR System (Applied Biosystems, Foster City, CA, USA) [36]. The primers and probes for each SNP were designed by Nanjing Steed BioTechnologies Co., Ltd (Table A in S1 File). The quality and potential misclassification of the genotyping results were assessed by evaluating 5% of duplicate DNA samples that were randomly selected from the whole samples. Their replicates were 100% concordant. The samples were tested blindly and genotyping results were judged by two researchers independently.

### Statistical analyses

Continuous variables were described as mean ± standard deviation (SD) or median (inter-quartile range, IQR) and differences between groups were analyzed by two-factor analysis of variance test or non-parametric test. Categorized variables were described as percentage and analyzed by χ^2^-test. Hardy-Weinberg equilibrium (HWE) was tested by a goodness-of-fit χ^2^-test. Haplotype blocks were selected by Haploview 4.2 software in consideration of the linkage disequilibrium (LD) between SNPs in each gene. The estimated frequency of polymorphic loci was calculated using PHASE 2.1 software. Odds ratios (OR) with 95% confidence intervals (CIs) was estimated by conditional logistic regression model. The statistical analyses were performed with SPSS for Windows (version 20.0, SPSS Inc., Chicago, IL, USA). Considering the potential false positive rate incurred by multiple comparisons of SNPs, we applied the Bonferroni correction method to adjust the P value. Sensitivity analysis was further performed to estimate the possible bias due to over-matching based on unmatched cases and controls.

Power calculation was performed for variant alleles using Quanto software package (Version 1.2.4, http://biostats.usc.edu/software). Case-to-control ratio of 1:4, gene only hypothesis, dominant inheritance model were used. We assumed a population risk of 0.12 for ATDH based on a systematic review [37] and considered a genetic relative risk (R_G_) of 2 with a two-sided p-value of 0.05.

## Results

### Baseline characteristics

The baseline characteristics of the 89 cases and 356 controls are summarized in Table 2. No significant difference was observed between the two groups regarding to demographic parameters, including age, weight or body mass index and baseline values of liver biochemical parameters (P>0.05). However, the peak AST, ALT, and total bilirubin levels of cases were significantly higher than controls during the treatment (P<0.0001). Of the 89 ATDH cases, 79 had over two times of ULN increased in ALT level and 10 combined an increase in AST and total bilirubin with at least one of them over two times of ULN.

**Table 2:** Characteristics of patients with and without ATDH.

Characteristic	Patients with ATDH (N = 89)	Patients without ATDH (N = 356)	P value
Sex (male/female) ^a^	65/24	260/96	1.000
Treatment history (primary/retreatment)	78/11	312/44	1.000
Age (years) ^a^	43.7 ± 16.4	43.6 ± 16.4	0.742
Weight (kg) ^a^	53.2 ± 8.0	53.5 ± 7.4	0.748
Body mass index (kg/m^2^) ^a^	19.5 ± 2.3	19.4 ± 2.3	0.959
Baseline value^b^			
AST(U/L) ^c^	24.8 (17.2–32.6)	21.4 (15.3–27.0)	0.283
ALT (U/L) ^c^	16.9 (10.8–26.3)	16.0 (10.4–22.0)	0.087
Total bilirubin (μmol/L) ^c^	9.5 (7.5–13.7)	9.7 (7.4–12.5)	1.000
During treatment			
Peak AST(U/L) ^c^	95.1 (60.6–174.7)	23.7 (16.7–29.0)	<0.0001
Peak ALT(U/L) ^c^	121.0 (88.2–183.6)	17.0 (11.6–23.2)	<0.0001
Peak total bilirubin (μmol/L)) ^c^	14.3 (11.4–18.0)	9.7 (6.8–13.7)	<0.0001

BMI, body mass index; ALT, alanine aminotransferase; AST, aspartate aminotransferase.

^a^ Values are presented as mean ± SD

^b^ Normal intervals: ALT<40 U/L, AST<40 U/L, Total bilirubin<19 umol/L.

^c^ Values are presented as median (inter-quartile range).

### IL-6, STAT3 and HSPA1L polymorphism in patients with and without ATDH

No deviation from Hardy-Weinberg equilibrium was observed for these seven polymorphisms in the controls (P >0.05) (Table 1). The genotype distributions of the selected SNPs in the case and control groups were presented in Table 3. The conditional logistic regression analysis revealed that no significant association was found between three IL-6 SNPs and ATDH. For STAT3 gene, rs1053023 was observed to associate with the risk of ATDH under an additive model, however this significance disappeared after Bonferroni correction. HSPA1L rs2227956 was also not associated with the risk of ATDH after Bonferroni correction (Table 3).

**Table 3:** IL-6, STAT3 and HSPA1L Polymorphisms in Patients with and without ATDH.

SNP	Cases (n = 89) N(%)	Controls (n = 356) N(%)	OR ^a^ (95% CI)	P value
IL-6 rs2066992 (G>T)				
GG	50 (56.2)	223 (63.0)	1 (reference)	
GT	29 (32.6)	112 (31.6)	1.19 (0.71–1.99)	0.508
TT	10 (11.2)	19 (5.4)	2.47 (1.06–5.76)	0.036
GT/TT	39(43.8)	131(37.0)	1.38 (0.86–2.21)	0.187
Additive			1.62 (0.95–2.76)	0.079
IL-6 rs2069837 (A>G)				
AA	57 (64.1)	244 (69.7)	1 (reference)	
AG	30 (33.7)	91 (26.0)	1.43 (0.86–2.37)	0.165
GG	2 (2.2)	15 (4.3)	0.62 (0.14–2.79)	0.530
AG/GG	32(35.9)	106(30.3)	1.32 (0.81–2.16)	0.267
Additive			1.15 (0.76–1.74)	0.504
IL-6 rs1524107 (C>T)				
CC	50 (56.2)	221 (62.6)	1 (reference)	
CT	30 (33.7)	112 (31.7)	1.23 (0.74–2.05)	0.432
TT	9 (10.1)	20 (5.7)	2.04 (0.87–4.82)	0.103
CT/TT	39(43.8)	132(37.4)	1.36 (0.85–2.18)	0.206
Additive			1.35 (0.94–1.93)	0.110
STAT3 rs1053004 (T>C)				
TT	38 (42.7)	138 (39.6)	1 (reference)	
TC	40 (44.9)	162 (45.9)	0.88 (0.53–1.46)	0.625
CC	11 (12.4)	53 (15.0)	0.77 (0.36–1.65)	0.502
TC/CC	51(57.3)	215(60.9)	0.86 (0.53–1.39)	0.532
Additive			0.88 (0.62–1.25)	0.474
STAT3 rs1053023 (A>G)				
AA	24 (27.0)	126 (35.6)	1 (reference)	
AG	40 (44.9)	167 (47.2)	1.27 (0.72–2.24)	0.403
GG	25 (28.1)	61 (17.2)	2.17 (1.15–4.11)	0.018
AG/GG	65(73.0)	228(64.4)	1.52 (0.90–2.56)	0.121
Additive			1.47 (1.06–2.04)	0.020
STAT3 rs1053005 (A>G)				
AA	40 (44.9)	155 (43.8)	1 (reference)	
AG	40 (44.9)	155 (43.8)	0.99 (0.61–1.62)	0.972
GG	9 (10.1)	44 (12.4)	0.83 (0.37–1.86)	0.650
AG/GG	49(55.1)	199(56.2)	0.96 (0.60–1.54)	0.861
Additive			0.94 (0.66–1.34)	0.722
HSPA1L rs2227956 (T>C)				
TT	36 (40.9)	195 (55.9)	1 (reference)	
TC	46 (52.3)	126 (36.1)	1.93(1.18–3.14)	0.008
CC	6 (6.8)	28 (8.0)	1.21(0.45–3.29)	0.708
TC/CC	52 (59.1)	154 (44.1)	1.83 (1.13–2.95)	0.013
Additive			1.43 (0.99–2.09)	0.060

^a^ Conditional logistic regression model analysis.

### Haplotype analysis of IL-6 and STAT3 in patients with and without ATDH

Linkage disequilibrium (LD) analysis was carried out on three SNPs of IL-6 and three SNPs of STAT3. In IL-6, strong LD between rs2069837, rs1524107 and rs2066992 was observed (D’>0.98, r^2^>0.74), thus we performed a haplotype analysis based on these three SNPs. In STAT3, strong LD between rs1053023, rs1053005 and rs1053004 was also observed (D’>0.95, r^2^>0.68). Results of haplotypes of these SNPs with risk of ATDH were listed in Table 4. The distribution of IL-6 haplotypes were similar in cases and controls. For gene STAT3, compared with those carrying rs1053023-rs1053005-rs1053004 AAT haplotype, those with rs1053023-rs1053005-rs1053004 GAT and AGC haplotypes were at a significantly increased risk of ATDH with ORs 12.38 (95% CI, 6.40–23.93, P<0.0001) and 20.81 (95% CI, 5.64–76.70, P<0.0001) respectively, after Bonferroni correction.

**Table 4:** ORs and 95% CIs for ATDH in relation to IL-6 and STAT3 haplotypes.

Haplotypes	Cases (%)	Controls (%)	OR (95% CI) ^a^	P value
IL-6 (rs2069837-rs1524107-rs2066992)				
ACG	129 (72.5)	559 (78.5)	1 (reference)	
GTT	34 (19.1)	120 (16.9)	1.26 (0.82–1.93)	0.299
GTG	14 (7.9)	30 (4.2)	2.09 (1.06–4.10)	0.032
STAT3 (rs1053023-rs1053005-rs1053004)				
AAT	78 (43.8)	410 (57.6)	1 (reference)	
GGC	48 (27.0)	238 (33.4)	1.06 (0.71–1.58)	0.866
GAT	38 (21.4)	28 (4.0)	**12.38 (6.40–23.93)**	**<0.0001** ^b^
GAC	4 (2.3)	25 (3.5)	0.83 (0.28–2.46)	0.702
AGC	10 (5.6)	4 (0.6)	**20.81 (5.64–76.70)**	**<0.0001** ^b^

^a^ Conditional logistic regression model analysis.

^b^ Significant after Bonferroni correction for multiple comparisons.

## Discussion

In this study, we tested the hypothesis that IL-6/STAT3 signaling pathway and the downstream HSP responsiveness may associate with the development of ATDH. Our result showed that although no significant difference in IL-6 and HSPA1L polymorphisms was observed between ATDH cases and controls, carriers of STAT3 rs1053023-rs1053005-rs1053004 GAT and AGC haplotypes were at a higher risk of ATDH, indicating that STAT3 might play a role in ATDH development.

Recent evidence from animal studies suggested that IL-6-deficient mice exhibit impaired liver regeneration characterized by liver necrosis, a blunted DNA synthesis response in hepatocytes, and reduced gene induction of the G1 phase [38]. By contrast, administration of IL-6 has been shown to serve key hepatoprotective functions against liver injury induced by Concanavalin A [39] and ischemia/reperfusion [40]. The relevance of these observations is still unclear in humans due to the rarity of studies. There was only one small case-control study involving 37 patients who suffered from transaminase elevation after tacrine administration and 32 controls treated with tacrine without experiencing hepatotoxicity, in which the authors investigated the role of IL-6 variants and tacrine-induced hepatotoxicity [27]. Another study in patients with a history of acetaminophen overdose found that levels of IL-6 were higher in patients with serum alanine aminotransferase > 1000 IU/L [41]. However, no other published studies have investigated the association between IL-6 polymorphisms and the risk of ATDH. Our study failed to find the significant association of IL-6 polymorphism with the risk of ATDH. The lack of significant association might be explained by the heterogeneity of culprit drugs and racial differences. In addition, some SNPs (such as rs1800796, rs1800795) in association with various diseases in Chinese population were not included, indicating that genotyping the cases and controls for other SNPs in IL-6 gene might be needed in the future.

As a member of signal transducers and activators of transcription (STATs) family, STAT3 mainly activated by IL-6 is a vital transcription factor providing hepatoprotection against Fas-mediated apoptotic liver damage by two mechanisms: direct inactivation of caspases and reduction of reactive oxygen species [18]. In the classical activation pathway, drug-induced stress and/or damage of hepatocytes may trigger inflammatory responses within the liver, thus it would produce a range of inflammatory mediators, including cytokines like IL-4, IL-6, IL-10 and TNF-∝. It is believed that the protective effect of IL-6 is mediated through binding to its soluble receptor sIL-6R, followed by dimerization of the gp130 protein and activation of the Janus kinase (JAK)-STAT3 signaling pathway. Dimerized STAT3 is able to translocate into the nucleus and activate gene transcription [12]. In addition, several STAT3 downstream genes including HSP70, Bcl-2, Bcl-xL, Mcl-1, FLIP, Ref-1, cyclin D1 and c-myc have been shown to have hepatoprotective effect [18,42].

Li [43] created liver-specific STAT3 deletion mice and reported that these mice exhibited reduced hepatocyte DNA synthesis and lower expression level of G1-phase cyclins, including cyclin D1 and E in the liver. From these results, they concluded that STAT3 accounts for at least part of cell cycle progression and cell proliferation during liver regeneration. Recently, a genome-wide association study in European descent found STAT4 (rs7574865) polymorphism associated with hepatocellular DILI, indicating the emerging role of STAT3 family in the etiology of DILI [44]. In our study, we found that carriers of STAT3 rs1053023-rs1053005-rs1053004 GAT and AGC haplotypes were at a higher risk of ATDH, however, our study on STAT3 AGC haplotype involved comparison of small groups of cases and controls which raised concerns about the observed low P value being simply change finding, therefore, this positive result needs to be confirmed in studies with larger sample size. Moreover, STAT3 could also be activated by several other factors including IL-22, IL-10, ECG and hepatitis viral proteins [45], suggesting that further well designed studies should be conducted to explore the association between them and the risk of ATDH.

Heat shock proteins (HSP) are produced by both prokaryotic and eukaryotic cells in response to a wide variety of stressful insults such as hypoxia, ischaemia, metabolic disruption and inflammatory mediators [46]. HSP70s perform housekeeping and quality controls in cells [47]. The first study to link hepatoprotective role of IL-6 and HSP70 expression was conducted by Masubuchi [19]. They found that the inducible HSP70 in the liver of IL-6^-/-^ mice was impaired when compared with wild-type mice following APAP treatment. In our study, for the purpose of replication in Chinese population, we only chose the reported SNP of HSPA1L gene (rs2227956), which is reported to be significantly associated with increased levels of serum HSP70[48,49],and we found that HSPA1L rs2227956 was not associated with the risk of ATDH after Bonferroni correction. The same polymorphism was found to be associated with Flucloxacillin-induced hepatitis, but it was attributed to its LD with nearby HLA-B genes (B*57:01 allele) [28], which was in part consistent with our finding. Therefore, further investigations focusing on HLA class I and II alleles might be needed in our population.

To the best of our knowledge, this is the first attempt to evaluate the association between variations in IL-6, STAT3 and HSPA1L genes and ATDH in Chinese Han population, which contributes to the further understanding of underlying mechanism in the pathogenesis of ATDH. In addition, our study was based on a well-designed prospective cohort which was the first population based study in China to investigate ADRs among TB patients during anti-TB treatment. All study participants were ethnic Chinese minimizing the potential confounding due to ethnics. Besides, only incident cases were included, thus it ruled out the possibility of recall and selection bias. All ATDH cases were carefully verified by experts which minimized the diagnosis misclassification.

There were also some limitations. Firstly, in our study, cases were matched with four controls based on sex, age at baseline (±5 years), treatment history, disease severity, drug dosage and place of sample collection, which may lead to the problem of over-matching. Therefore, we did a sensitivity analysis based on unmatched cases and controls and no major difference from the main analyses was observed (Table B and C in S1 file). Another limitation is the small sample size, which is because some patients with HBsAg (+), alcohol drinking, liver diseases, and so on were excluded from this study. However, in our study, the statistical power for the given sample size demonstrated that all the analyses were not underpowered (above 80%), indicating that the sample size in our study was adequate to detect the association between the selected SNPs and ATDH (Table D in S1 file). In addition, we did not test for HCV infection, so we cannot rule out the possible confounding, although the prevalence of HCV infection is very low in the study population [50] and we did take the participants’ history of liver disease into consideration. Genetic associations were ethnicity-specific, indicating that a significant association between a specific drug and a specific adverse reaction may not generalize from one ethic group to another. Thus, this positive association should be re-examined in other ethnics.

## Conclusion

This study suggested that genetic variants of STAT3 might contribute to ATDH susceptibility in Chinese Han population. Studies in larger, varied populations are required to confirm these findings.

## Supporting Information

S1 File Contains the following files. **Table A.** Information of primers and probes. **Table B.** IL-6, STAT3 and HSPA1L Polymorphisms in Patients with and without ATDH (unmatched case-control study). **Table C.** ORs and 95% CIs for ATDH in relation to IL-6 and STAT3 haplotypes (unmatched case-control study). **Table D.** Power calculation results.
(DOCX)
