# Supplementary material for: Analysis of IL-6, STAT3 and HSPA1L Gene Polymorphisms in Anti-Tuberculosis Drug-Induced Hepatitis in a Nested Case-Control Study
Source: PLoS One. 2015 Mar 19;10(3):e0118862. doi: 10.1371/journal.pone.0118862 (PMC4366259; doi:10.1371/journal.pone.0118862)
Supplement: S1 File — Table A. Information of primers and probes. Table B. IL-6, STAT3 and HSPA1L Polymorphisms in Patients with and without ATDH (unmatched case-control study). Table C. ORs and 95% CIs for ATDH in relation to IL-6 and STAT3 haplotypes (unmatched case-control study). Table D. Power calculation results. (DOCX) [file pone.0118862.s001.docx]

**Table A.** Information of primers and probes

| SNPs | Primer sequence (5’-3’) | Probe sequence |
| --- | --- | --- |
| rs2066992  G>T | F-GATGCCAATGAGTTGTAGCTTCA  R-GTGTCCCTAAAACAAACACCACTAGA | G:FAM-AACAATGAAAAGGCC -MGB  T: HEX-ACAATGAAAAGTCC-MGB |
|  |  |  |
| rs2069837  A>G | F-GCTGGAACATTCTATGGCTTGAA  R-TCTCCAAAAACCTTCCTTGCA | G: FAM-AGGCACTTTAGATAAA -MGB  A: HEX-CCAGGCACTTTAAATA -MGB |
|  |  |  |
| rs1524107  C>T | F-GATGCCAATGAGTTGTAGCTTCA  R-TGTCCCTAAAACAAACACCACTAGAG | T: FAM-TTTTCTTAGAGATTTTCCTGGC-MGB  C: HEX-TTTTCTTAGAGACTTTCC -MGB |
|  |  |  |
| rs1053004  T>C | F-CCACAGGTGAGGCAGAACAG  R-GAGTCAGAGGCAGCCCATC | C: FAM-CTAGCTCGCCTCTC-MGB  T: HEX-CTAGCTTGCCTCTC-MGB |
|  |  |  |
| rs1053023  A>G | F-CCTTAGGTAGCATGTATCTGGTCTT  R-GGGGCAGTGGACAGGAAG | A: FAM-TGGCCCATTAAAG-MGB  G: HEX-TGGCCCGTTAAAG-MGB |
|  |  |  |
| rs1053005  A>G | F-GAGTTTCTGTGGAATTCTGTTTGTTAA  R-CTCTGCATGACACATCAACTGTCT | G: FAM-AGACGACCTTCTCTAGGA-MGB  A: HEX-ACCTTCTCTAAGATGAACA-MGB |
|  |  |  |
| rs2227956 | F-TGATGCCAATGGTATTCTCAATG | T: FAM-CACAGCCATGGACA-MGB |
| T>C | R-CAGGCGGCCCTTGTCAT | C: HEX-CACAGCCACGGAC-MGB |

**Table B.** IL-6, STAT3 and HSPA1L Polymorphisms in Patients with and without ATDH (unmatched case-control study)

| SNP | Cases  (n=89)  N(%) |  | Controls (n=356)  N(%) | OR ^a^  (95% CI) | P value |
| --- | --- | --- | --- | --- | --- |
| IL-6 rs2066992 (G>T) | | | | |  |
| GG | 50 (56.2) |  | 223 (63.0) | 1 (reference) |  |
| GT | 29 (32.6) |  | 112 (31.6) | 1.16 (0.69-1.92) | 0.581 |
| TT | 10 (11.2) |  | 19 (5.4) | 2.35 (1.03-5.36) | 0.043 |
| GT/TT | 39(43.8) |  | 131(37.0) | 1.33 (0.83-2.13) | 0.238 |
| Additive |  |  |  | 1.38 (0.96-1.97) | 0.084 |
| IL-6 rs2069837 (A>G) | | | | |  |
| AA | 57 (64.1) |  | 244 (69.7) | 1 (reference) |  |
| AG | 30 (33.7) |  | 91 (26.0) | 1.41 (0.85-2.33) | 0.180 |
| GG | 2 (2.2) |  | 15 (4.3) | 0.57 (0.13-2.57) | 0.571 |
| AG/GG | 32(35.9) |  | 106(30.3) | 1.29 (0.79-2.11) | 0.304 |
| Additive |  |  |  | 1.12 (0.74-1.69) | 0.580 |
| IL-6 rs1524107 (C>T) | | | | |  |
| CC | 50 (56.2) |  | 221 (62.6) | 1 (reference) |  |
| CT | 30 (33.7) |  | 112 (31.7) | 1.18 (0.71-1.97) | 0.514 |
| TT | 9 (10.1) |  | 20 (5.7) | 1.99 (0.86-4.63) | 0.110 |
| CT/TT | 39(43.8) |  | 132(37.4) | 1.31 (0.82-2.09) | 0.267 |
| Additive |  |  |  | 1.31 (0.92-1.89) | 0.138 |
| STAT3 rs1053004 (T>C) | | | | |  |
| TT | 38 (42.7) |  | 138 (39.6) | 1 (reference) |  |
| TC | 40 (44.9) |  | 162 (45.9) | 0.90 (0.55-1.48) | 0.668 |
| CC | 11 (12.4) |  | 53 (15.0) | 0.75 (0.36-1.58) | 0.455 |
| TC/CC | 51(57.3) |  | 215(60.9) | 0.86 (0.54-1.38) | 0.535 |
| Additive |  |  |  | 0.88 (0.62-1.23) | 0.446 |
| STAT3 rs1053023 (A>G) | | | | |  |
| AA | 24 (27.0) |  | 126 (35.6) | 1 (reference) |  |
| AG | 40 (44.9) |  | 167 (47.2) | 1.26 (0.72-2.19) | 0.420 |
| GG | 25 (28.1) |  | 61 (17.2) | 2.15 (1.14-4.07) | 0.019 |
| AG/GG | 65(73.0) |  | 228(64.4) | 1.50 (0.89-2.51) | 0.126 |
| Additive |  |  |  | 1.46 (1.06-2.02) | 0.022 |
| STAT3 rs1053005 (A>G) | | | | |  |
| AA | 40 (44.9) |  | 155 (43.8) | 1 (reference) |  |
| AG | 40 (44.9) |  | 155 (43.8) | 1.00 (0.61-1.64) | 1.000 |
| GG | 9 (10.1) |  | 44 (12.4) | 0.79 (0.36-1.76) | 0.568 |
| AG/GG | 49(55.1) |  | 199(56.2) | 0.95 (0.60-1.52) | 0.844 |
| Additive |  |  |  | 0.93 (0.66-1.31) | 0.665 |
| HSPA1L rs2227956 (T>C) | | | | |  |
| TT | 36 (40.9) |  | 195 (55.9) | 1 (reference) |  |
| TC | 46 (52.3) |  | 126 (36.1) | 1.98 (1.21-3.23) | 0.006 |
| CC | 6 (6.8) |  | 28 (8.0) | 1.16 (0.45-3.00) | 0.759 |
| TC/CC | 52 (59.1) |  | 154 (44.1) | 1.83 (1.13-2.94) | 0.013 |
| Additive |  |  |  | 1.39 (0.97-1.99) | 0.071 |
| ^a^ Unconditional logistic regression model analysis. | | | | | |

**Table C.** ORs and 95% CIs for ATDH in relation to IL-6 and STAT3 haplotypes

(unmatched case-control study)

| Haplotypes | Cases  (%) | Controls  (%) | OR (95% CI) ^a^ | P value |
| --- | --- | --- | --- | --- |
| IL-6 (rs2069837-rs1524107-rs2066992) | | | |  |
| ACG | 129 (72.5) | 559 (78.5) | 1 (reference) |  |
| GTT | 34 (19.1) | 120 (16.9) | 1.26 (0.82-1.93) | 0.298 |
| GTG | 14 (7.9) | 30 (4.2) | 2.18 (1.11-4.26) | 0.023 |
|  |  |  |  |  |
| STAT3 (rs1053023-rs1053005-rs1053004) | | | |  |
| AAT | 78 (43.8) | 410 (57.6) | 1 (reference) |  |
| GGC | 48 (27.0) | 238 (33.4) | 1.08 (0.73-1.60) | 0.696 |
| GAT | 38 (21.4) | 28 (4.0) | **7.15 (4.14-12.34)** | **<0.0001** ^b^ |
| GAC | 4 (2.3) | 25 (3.5) | 0.86 (0.29-2.55) | 0.786 |
| AGC | 10 (5.6) | 4 (0.6) | **14.12 (4.30-46.40)** | **<0.0001** ^b^ |
| ^a^ Unconditional logistic regression model analysis.  ^b^ Significant after Bonferroni correction for multiple comparisons | | | | |

**Power Calculation:**

We made the following assumptions:

1. **Design:** case-control (1:4)
2. **Hypothesis:** Gene Only
3. **Sample size:** 89 cases, 4 controls per case are required
4. **Significance:** 0.05, 2-sided
5. **Model of inheritance:** Dominant
6. **The prevalence of the disease in the population (K_p_):** 12%
7. **The relative risk for risk allele carriers (R_G_):** 2.00

**Table D.** Power calculation results

| Gene | SNP No. | Base Change | Allele Frequency ^a^ (%) | R_G_ ^b^ | Power |
| --- | --- | --- | --- | --- | --- |
| IL-6 | rs2066992 | G>T | 24.4% | 2.00 | 82.8% |
| IL-6 | rs2069837 | A>G | 19.8% | 2.00 | 82.2% |
| IL-6 | rs1524107 | C>T | 24.4% | 2.00 | 82.8% |
| STAT3 | rs1053004 | T>C | 32.6% | 2.00 | 80.3% |
| STAT3 | rs1053023 | A>G | 27.9% | 2.00 | 82.2% |
| STAT3 | rs1053005 | A>G | 26.7% | 2.00 | 82.5% |
| HSPA1L | rs2227956 | T>C | 18.6% | 2.00 | 81.7% |
| ^a^ Allele frequency for Han Chinese in Beijing in NCBI dbSNP (<http://www.ncbi.nlm.nih.gov/projects/SNP>).  ^b^ R_G_: the relative risk for risk allele carriers | | | | | |
